# Elbow Ulnar Collateral Ligament Reconstruction Using the Novel Docking Plus Technique in 324 Athletes

**DOI:** 10.1186/s40798-018-0174-8

**Published:** 2019-01-16

**Authors:** Benjamin F. Donohue, Marc G. Lubitz, Timothy E. Kremchek

**Affiliations:** 1 Cayuga Medical Associates, 16 Brentwood, Suite A, Ithaca, NY 14850 USA; 2 UMass Medical School, 55 Lake Ave N, Worcester, MA 01655 USA; 3 Beacon Orthopeadics and Sports Medicine, 500 E Business Way, Cincinnati, OH 45241 USA

**Keywords:** UCL, Elbow, Baseball, Tommy John Surgery, Ligament reconstruction

## Abstract

**Background:**

This retrospective case series examined 324 athletes who received elbow ulnar collateral ligament (UCL) reconstruction by a single surgeon in a private practice over a 9-year period. The novel *Docking Plus* technique for elbow UCL reconstruction in 324 athletes provided good or excellent Conway score results in 88% of patients. The preponderance of previous studies examining UCL reconstruction outcomes were performed by surgeons at one of only three institutions (Andrews Institute, Hospital for Special Surgery, Kerlan Jobe Orthopedic Clinic).

**Methods:**

Patients undergoing UCL reconstruction from November 2005 to December 2014 were identified and contacted with a mailed survey and phone call. These patients were given a subjective 19 question survey assessing their outcomes from surgery.

**Results:**

The participants who responded to our survey were 90% male and 77% baseball players, 73% of which were pitchers. Of the baseball players who responded, 51.9% were in high school at the time of their surgery, 37% college, 6.5% minor leagues, and 2.2% in Major League Baseball. After surgery, 36% of survey responders returned to a higher level of competition than previously. For example, a high school athlete who had UCL reconstruction and went on to pitch in college. Further, 45% returned to the same level, and 7% returned to a lower level. Subjective “satisfaction,” was reported in 92% of responders and 97.2% reported that, “having surgery was a good idea.” Symptom onset in the responding athletes was 58.9% sudden, and 41.1% gradual. Overall, 90.9% of respondents returned to play in less than 1.5 years while 6.3% never were able to return. Re-tear occurred in 2.5% of patients, while 8.8% had subjective nerve dysfunction for at least 3 months following surgery.

**Conclusion:**

The Docking Plus technique can produce excellent subjective and objective results in athletes. Further study is warranted to see the effects of this procedure in other settings and determine which method of reconstruction or repair is superior.

## Key Points

- The majority of UCL reconstruction outcomes studies are from just three major institutions
- Eighty-eight percent of survey respondents who completed their rehab protocols following UCL reconstruction with the Docking Plus technique had a Conway-Jobe score of good or excellent
- More studies examining objective outcomes following UCL reconstruction are needed

## Background

Injury to the elbow ulnar collateral ligament (UCL) and its surgical reconstruction in throwing athletes is well described [2, 4, 5, 7, 8, 10–26, 29, 32–36, 41, 43, 47, 60, 64, 74]. The incidence of UCL reconstruction surgery is increasing, among high school athletes and professional athletes [6, 17, 35, 46, 77, 79].

The UCL is the primary valgus stabilizer of the elbow. It experiences the most torque in the late cocking and early acceleration phases of the pitching motion kinetic chain [38, 40, 41, 58, 59, 75]. Pitching velocity and volume have both been associated with an increased risk of UCL injury [3, 14, 28, 31, 39, 52, 61, 62, 67, 78]. 

Patients often, though not always, complain of acute or chronic medial elbow pain when throwing, which affects velocity and accuracy. Physical exam generally reveals pain with elbow valgus stress testing and palpation of the UCL [45]. MRI with intra-articular contrast and dynamic ultrasound have the highest sensitivity in diagnosis of UCL injuries [20, 37, 71, 76]. Reconstructive surgery is typically recommended for full-thickness tears as well as partial thickness tears refractory to non-operative management. Internal bracing for partial tears has recently emerged as a possible alternative to full reconstruction [30].

Since the year 2000, published case series of surgical outcomes for UCL reconstruction have demonstrated rates of return-to-play (RTP) of 74–100% [4, 10, 13, 19, 21, 22, 24, 29, 49, 50, 63, 66, 69, 72]. Studies of Major League Baseball (MLB) pitchers have shown RTP rates of 67–87% [34, 44, 50, 53, 56].

A variety of surgical techniques have been described, including the Docking Plus technique [57]. Though UCL reconstruction patient outcomes have been measured in multiple level 3 and 4 studies, these studies have typically had low numbers (< 100) of patients. Also, the preponderance of the included surgeries were performed by surgeons at one of only three institutions (Andrews Institute, Hospital for Special Surgery, Kerlan Jobe Orthopedic Clinic). The purpose of this paper is to provide data from previously unreported outcomes of 324 athletes at a single surgeon center who underwent UCL reconstruction using the “Docking Plus,” technique.

## Methods

We identified all UCL reconstructions performed by the senior author (TEK) from November 2005 to December 2014. We did so by searching the senior surgeon’s practice patient database by surgeon and Current Procedural Terminology (CPT) code (24346). CPT code 24346 is defined as, “Reconstruction medial collateral ligament, elbow, with tendon graft (includes harvesting of graft)”. This yielded 655 results of 647 unique patients. These reconstructions were all done with the docking plus technique and utilized the contralateral palmaris longus tendon for the graft when present. If the patient did not have palmaris longus tendons, a gracilis tendon autograft was used.

A literature search was conducted to compare our study’s outcomes to previously reported data. We searched PubMed and Google Scholar for UCL reconstruction outcome studies and found 25 such studies published between 1986 and 2014 [4, 8, 10, 13, 19, 21, 22, 25, 29, 34, 44, 48–51, 53, 55, 56, 63, 65, 66, 69, 70, 72]. Of these studies, 19 [4, 8, 10, 13, 19, 21, 22, 25, 29, 49, 51, 54, 55, 63, 65, 66, 69, 70, 72] detailed outcomes by surgeon authors, while 6 studies [34, 44, 48, 50, 53, 56] reported outcomes with data from MLB databases.

For each study, we charted the outcomes and patient and injury characteristics measured. All of the 19 case series measured the competition level of return-to-play with 12 of these studies explicitly using Conway-Jobe or modified Conway-Jobe criteria in their classifications. Several other outcome metrics were used in these case series, including the Andrews-Timmerman elbow score (3 of 19 studies) [22, 51, 55], the KJOC (Kerlan Jobe Orthopedic Clinic) Score (1 of 19) [51], DASH (Disabilities of the Arm, Shoulder, & Hand) score (1 of 19) [63], and MEPI (Mayo Elbow Performance Index) score (1 of 19) [54].

A 2-page, 19-question survey was designed with questions about demographics (e.g., age, gender), sports participation (e.g., sport, position, handedness), the nature of the injury (e.g., speed of onset, timing, prior surgery), subjective outcomes (e.g., patient satisfaction), and objective outcomes (e.g., timing and level of return-to-play, Conway-Jobe score, career longevity, reasons for retirement, complications).

We used contact information (mailing address, telephone number) provided by patients at the time of prior clinical encounters to contact the patients. We mailed letters containing a URL link to the survey and a unique, anonymized patient identification number. In addition to standard mail, we contacted patients by telephone to provide them with the survey URL, their identification numbers, and to briefly describe the purpose of the outcomes study. We obtained an advisory opinion which states that our survey posed minimal risk and met criteria for being IRB exempt.

### Surgical Technique

**Fig. 1:**
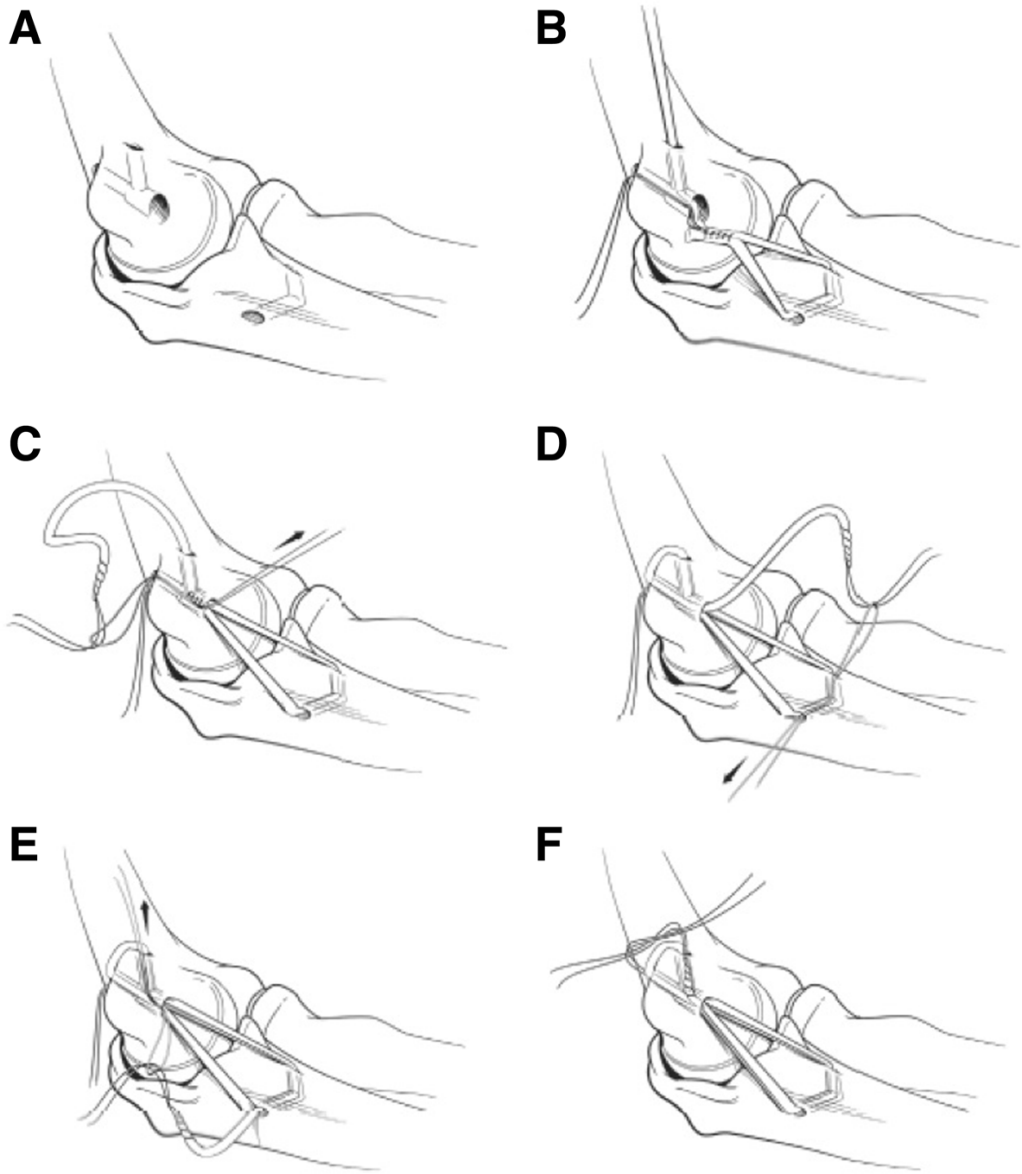
The Docking Plus Technique. **a** The bone tunnel locations within the proximal ulna and medial epicondyle of the humerus. **b** The graft is first passed through the ulnar tunnel, and the short end of the graft is sutured in a Krackow fashion; then, the suture ends are brought out the posterior exit holes of the medial epicondyle, while the graft is taken through the anterior exit holes. **c** The long end of the graft is then passed through the posterior medial epicondyle tunnel as the sutures are held at constant tension. **d** The graft is passed once again through the ulnar bone tunnels. **e** For the final pass, the graft is taken through the longitudinal tunnel of the medial epicondyle and out the anterior exit hole opposite the tensioned short end of the graft. **f** Both ends of the graft were then tensioned and tied together as the arm was held in a reduced position with no valgus stress at approximately 30° of flexion and the forearm in neutral rotation. (McGraw et al. 2013)

**Fig. 2:**
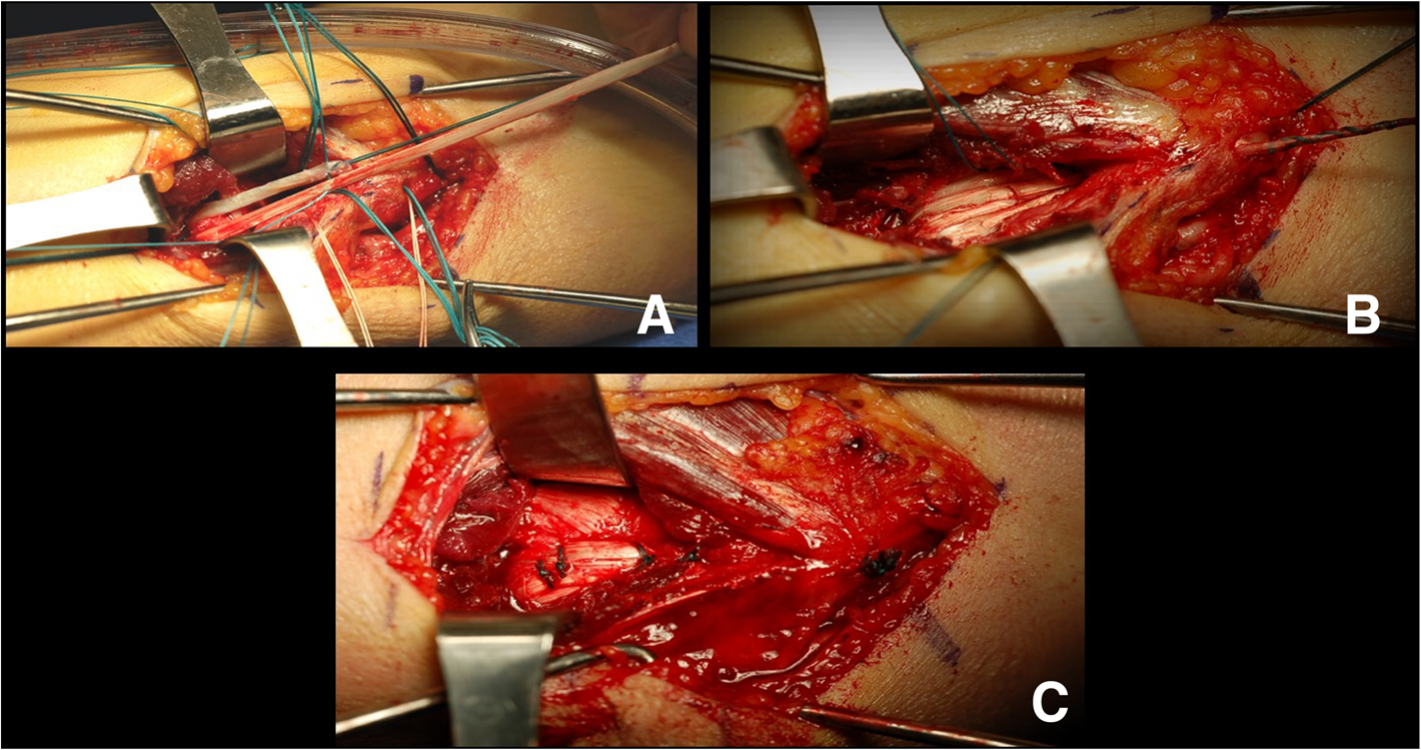
Intraoperative photos. **a** Krakow stitch placement between short and long limbs, **b** tensioning of the 4-strand graft, and **c** figure-4 stitches placed between the 4-strands of the reconstruction and the remnant native UCL ligament

The Docking Plus technique for UCL reconstruction (Figs. 1 and 2) was utilized. The patient is positioned supine with the operative extremity on a hand table. They are prepped and draped in sterile fashion. The elbow is flexed to 30° and a curved incision is made just posterior to the medial epicondyle. Care is taken to avoid the medial antebrachial cutaneous nerve during dissection, and the ulnar nerve is visualized to allow protection during retraction. The nerve is not routinely transposed. The flexor mass is split and the native UCL is identified. The UCL is then incised and peeled back. A 2.7-mm drill is used to make two unicortical drill holes, one anterior and one posterior to the sublime tubercle. The holes are curetted until they converge and a passing suture is passed through. A unicortical socket near the humeral anatomic origin of the UCL is made with a 4-mm burr. A 2.7-mm drill is used to make two unicortical holes on the posterior medial epicondyle. A curette is then used to connect these tunnels to the socket and suture is passed between the socket and one of the holes in the medial epicondyle. The graft is then passed through the two holes in the ulna. The short arm of the graft, the docking end, is aligned with the long arm of the graft and they are sutured together. The long arm is then passed out the lateral medial epicondyle tunnel, then the medial tunnel, across the ulnohumeral joint, through the ulnar holes, and back across the ulnohumeral joint and out the lateral hole in the epicondyle. The graft is tensioned as the arm is flexed and extended. The elbow is flexed to 70° in neutral rotation and the sutured from the graft is tied. The four graft strands which cross the ulnohumeral joint are sutured together. The wound is then closed. This technique uses longer strands of autograft when compared to more commonly used techniques like the Standard Docking. This allows for two extra passes of the graft over the medial epicondyle and more graft to bone surface area for healing. Palmaris longus or contralateral gracilis tendons were used for autograft.

Postoperative rehabilitation utilized standard timelines of immobilization, strengthening, throwing programs, and return-to-play [9, 42, 68]. In weeks 1–2, evaluation of ulnar nerve function is critical. This is monitored closely by the physical therapist and the surgeon is made aware of any possible deficit. The elbow is passively ranged from 30 to 90°, which can be increased if tolerated. Extension is dependent and posterior pinching. Active range of motion (AROM) at the wrist is encouraged and exercises involving grip and hand strength are performed. Cryotherapy and electrical stimulation are also utilized around the elbow. The patients brace is left locked at 60°. Sutures are removed at 10–14 days. At 3 weeks, the brace is set at 30–90° following approval by the surgeon. Isometric wrist and forearm exercises are begun. Elbow flexion and extension isometrics are started. AROM advanced to 20–105° if tolerating. At week 4, the brace is set at 15–105° and they are passively ranged from 10 to 120. Hand intrinsic muscle therapy is progressed and cardiovascular conditioning on a stationary bike is started. In weeks 5–6, the brace is fully opened and taken off by the end of week 6. Passive range of motion (PROM) is increased to 0–130°. Wrist and elbow resistance exercises with 1 lb of weight are begun. A shoulder strengthening program is started.

## Results

Of the 647 patients who received a survey, 3 were excluded because they did not undergo UCL reconstruction and 2 because they underwent revision UCL reconstruction. We received 335 responses to the survey, although 9 were found to be duplicate responses, 1 patient failed to respond with either his survey ID number or name, and 1 patient responded with a nonsense ID number. Therefore, we analyzed 324 responses for a 50.1% response rate. These patients had all undergone UCL reconstruction using the “Docking Plus” technique.

Survey responders were 90% (290 of 324) male, while 10% (34 of 324) were female.

Baseball was the primary sport for 77% (248) of responders. Other primary sports included softball (13), football (14), wrestling (9), volleyball (6), gymnastics (5), golf (5), cheerleading (4), tennis (4), lacrosse (4), rugby (1), and track and field (1).

**Fig. 3:**
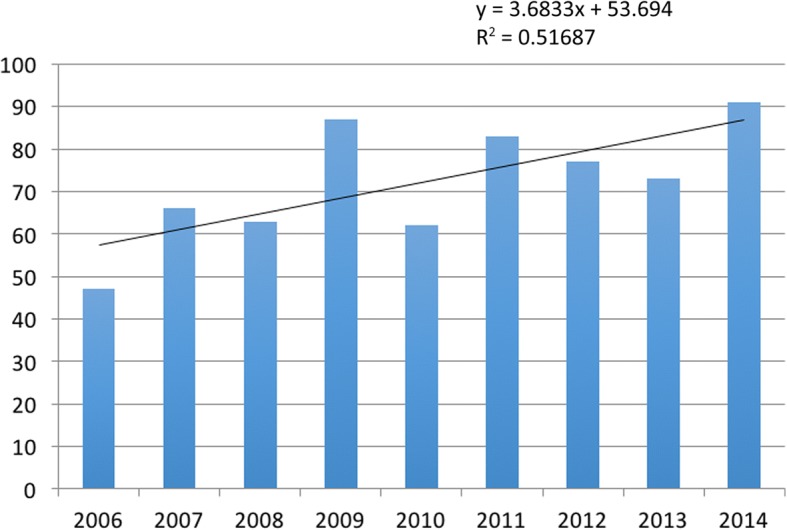
Number of UCL reconstructions performed per year from 2006 to 2014

Of the baseball players, 73% were pitchers, 9% catchers, 12% infielders, and 7% outfielders (Fig. 3).

Prior to surgery, the level of performance for all athletes was middle school (2.5%), high school (51.9%), college (37.0%), minor league (6.5%), and major league (2.2%). After surgery, the highest level of performance reached was middle school (1.9%), high school (27.2%), college (54.9%), minor league (14.2%), or major league (1.9%).

**Fig. 4:**
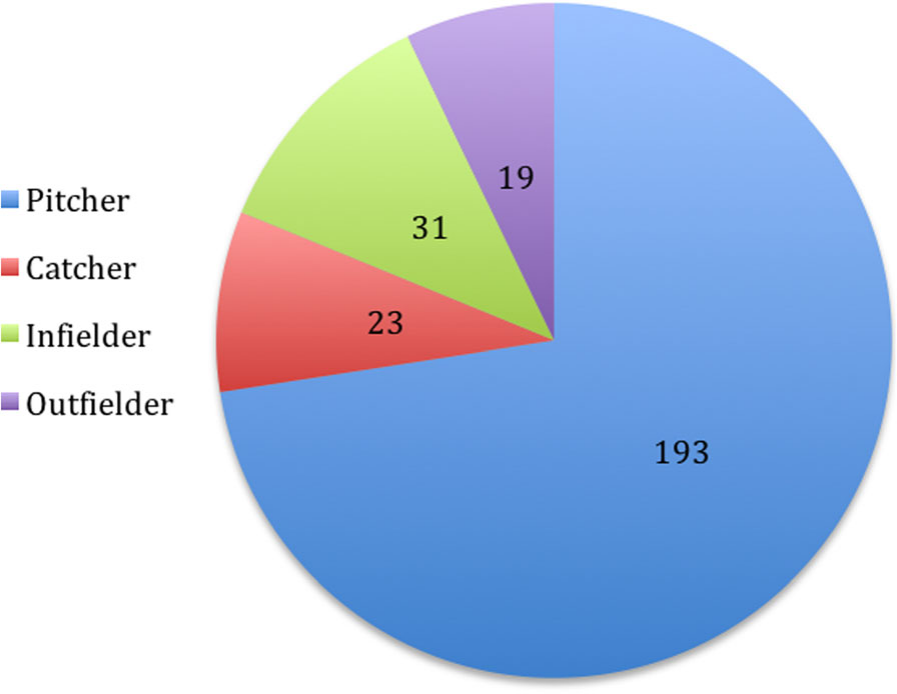
UCL reconstruction patients by position

**Fig. 5:**
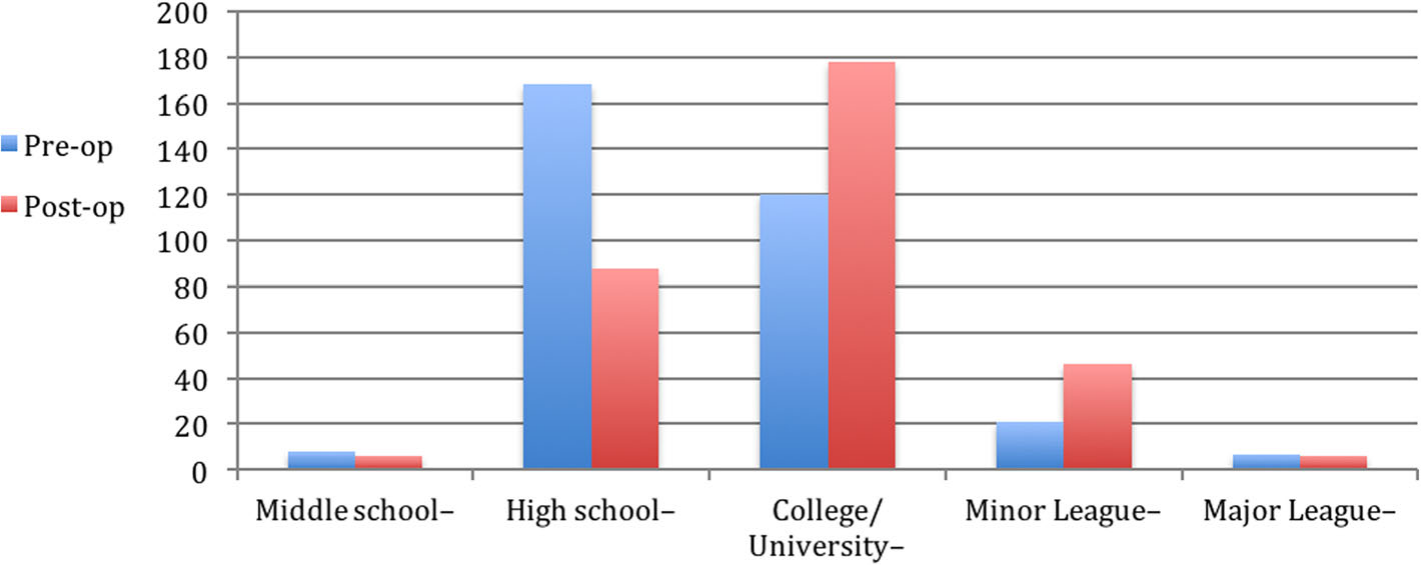
Level of sports competition

When asked explicitly about the level of return-to-play postoperative compared with preoperatively, 36% (104) had returned to a higher level of competition for at least one season, 45% (130) had returned to the same level, and 7% (20) had returned to a lower level. Thus, 81% had a Conway-Jobe score of excellent, and 88% had a score of excellent or good. Note that only 88.3% (286) of all respondents had completed their rehabilitation regiment and so were eligible to answer this question (Figs. 4 and 5).

**Fig. 6:**
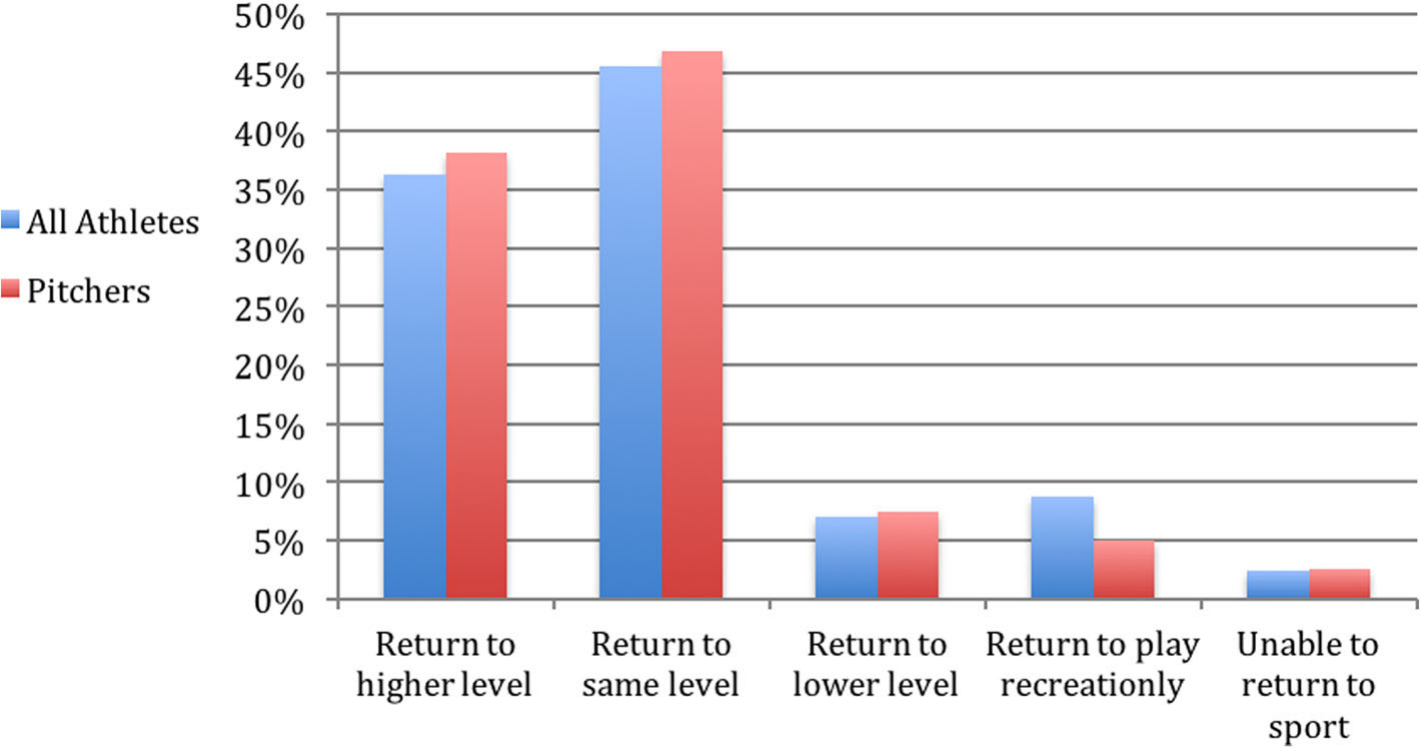
Level of return to play postoperatively

Subjectively, 92.9% (301 of 324) were satisfied with their surgery and 97.2% (315 of 324) thought that it was a good decision to have the surgery (Fig. 6).

While patients described pre-op symptom duration and non-operative management for as little as 0–3 months and as long as greater than 2 years, 43.3% (138) had symptoms for 0–3 months and 68.1% (194) underwent physical therapy for 0–3 months.

Symptom onset was sudden in 58.9% (188), but insidious/gradual in 41.1% (131). Of baseball pitchers, the symptom onset was sudden in 50.5% (93), but insidious in 49.5% (91). Then, 7.2% (23) had undergone prior surgery on the same elbow.

RTP timing varied from less than 9 months to over 2 years. Further, 90.9% (259) returned in less than 1.5 years, while 2.5% (7) returned in 1.5–2 years, 0.4% (1) returned in over 2 years, and 6.3% (18) did not return to play. Among pitchers, self-reported return-to-play was similar, with less than 9 months (7%, 11), 9–12 months (48%, 75), 1–1.5 years (33%, 52), 1.5–2 years (3%, 5), > 2 years (1%, 1), and never (8%, 13).

Complications included UCL re-tear in 2.5% (8) and additional same-side elbow surgery for any reason in 5.6% (18). Then, 8.8% (28) described some element of nerve dysfunction for at least 3 months postoperatively, although the survey did not distinguish between transient or permanent incision site numbness and ulnar nerve dyesthesia.

In baseball pitchers, there was self-reported subjective improvement in pitch velocity, control, ERA, and innings pitched as compared with pre-injury. Of the 155 who have by now discontinued playing their sport, only 14.8% (23) state that retirement was caused by elbow disability.

## Discussion

There are many UCL reconstruction outcome studies in published literature. However, most are produced by the same few pioneering institutions, such as the Andrews Institute [4, 13, 29, 63, 66, 69], Hospital for Special Surgery [10, 22, 24, 50, 69], and Kerlan Jobe Orthopedic Clinic [19, 21, 49, 72]. This raises questions as to whether or not the results can be extrapolated to other centers. This study was also performed at only one center by one surgeon, raising similar questions. However, any addition to the pool of data regarding UCL reconstruction outcomes can help the community direct future goals and studies. This study also likely represents the largest case series of women (34 athletes) who have undergone UCL reconstruction. It is the only case series published on patients who have had UCL reconstruction with the “Docking Plus” technique.

**Fig. 7:**
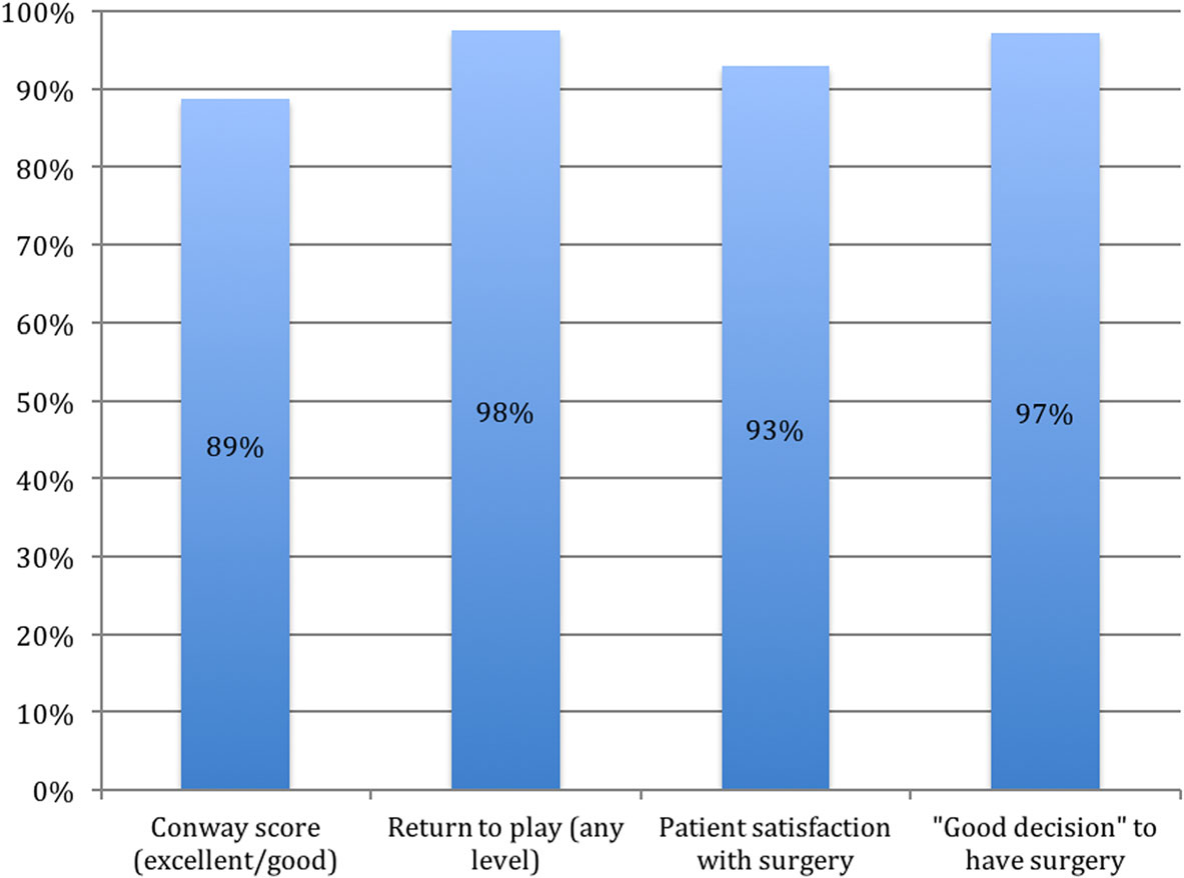
Patient outcome measures

Despite the increasing incidence of UCL reconstruction surgery (Fig. 7), only several outcome studies have contained more than 100 patients [10, 29, 63, 70], almost all of those on the patients of Dr. James Andrews [10, 29, 63], limiting the applicability of the results.

The Docking Plus technique, a variation on previously described Modified Docking techniques, has been previously described [27] and tested biomechanically [16], though outcome studies have not yet validated its use.

The goal of this study was to assess the performance of the Docking Plus technique of UCL reconstruction done by a single surgeon outside of the aforementioned pioneering institutions in a high volume patient series. The combined Conway score of good or excellent results of 88% is consistent with the results of similar outcome studies (Table 1).

**Table 1:** Conway scores by study

Year	Authors	*N*	Excellent/Good
2014	Osbahr et al. [63]	256	83%
2013	Jones et al. [50]	55	87%
2013	Savoie et al. [70]	116	93%
2012	Dines et al. [22]	10	90%
2012	Dugas et al. [29]	120	90%
2012	Kodde et al. [54]	20	90%
2010	Cain et al. [13]	743	86%, 91%*
2010	Bowers et al. [10]	21	100%
2007	Dines et al. [21]	22	86%
2006	Dodson et al. [25]	100	97%
2006	Paletta and Wright [65]	25	92%
2006	Koh et al. [55]	19	95%
2004	Petty et al. [66]	27	74%
2002	Rohrbough et al. [69]	36	92%
2001	Thompson et al. [72]	33	94%
2000	Azar et al. [8]	78	81%*
1994	Andrews and Timmerman [4]	9	na
1992	Conway et al. [19]	56	83%
1986	Jobe et al. [49]	16	69%

Subjective patient satisfaction scores in this study indicate that almost 96% patients think that it was a good decision for them to have surgery. Nearly as many, 92.9%, were satisfied with their surgery. This compares very favorably to patient satisfaction information from a study by Conte et al. in 2015 that found that only 72% of professional baseball players would have the surgery again and 17% would not have the surgery [24].

Our data regarding symptom onset (59% acute, 41% gradual) varies from those results previously published in the literature that are closer to a 1:1 incidence of these two presentations [34]. Though, in baseball pitchers, our symptom onset (51% acute, 49% gradual) was consistent with the reported incidences. Even when the presentation is described as “acute” or “traumatic” in throwing athletes, it is likely that there have been some underlying chronic degenerative changes to the UCL.

Time to return-to-play varies based on position and sport. There has been debate about delaying further professional pitchers timing of return-to-play based on some trends in improved pitching in the second year compared with in the first year after return-to-play [69, 72].

The rehabilitation protocol for pitchers dictates a return-to-play at 11–14 months if there are no setbacks. Eighty-one percent of these studies pitchers returned to pitch at 9–18 months postoperative. This is consistent with surgeon outcome studies that have shown a 11–13 month average return-to-pitch timing [10, 14, 15, 73].

Outcome studies that have utilized information from MLB databases have shown a mean return-to-play timing of 16–20 months [1, 69, 72, 78]. However, analysis of MLB data by a journalist have demonstrated that median return-to-play times in this population since 2002 have been consistently 13 months [79]. It seems that a few extended and complicated postoperative courses in MLB pitchers have skewed up the means reported in academic studies. Return-to-play is faster for baseball position players and non-baseball players.

### Limitations

The data in this study was self-reported and so is vulnerable to recall bias. Electronic medical records were accessed for the date of surgery and CPT code, but not for details of the patients’ histories and physical exams.

Return to sport can be affected by a variety of social and medical factors unrelated to the technical success of the surgical procedure including continued interest in sport, skill level, and commitment to a lengthy rehabilitation process. As much as the senior surgeon’s patient selection includes operating on patients who have the interest and ability to continue competing, these variables may change over time.

While a patient response rate of 50.1% of consecutive patients compares favorably with existing literature on UCL surgery outcomes, it is low enough to introduce significant selection bias into the results, especially those regarding patient satisfaction.

## Conclusion

The Docking Plus technique, performed in a private practice setting outside of the previously mentioned UCL reconstruction pioneering hospitals, produces excellent subjective (e.g., patient satisfaction) and objective (e.g., Conway score) results. Further study is warranted to see if these results can be extrapolated to other surgeons and patient populations.

## Abbreviations

AROM: Active range of motion
DASH: Disabilities of the Arm Shoulder & Hand
KJOC: Kerlan Jobe Orthopedic Clinic
MEPI: Mayo Elbow Performance Index
MLB: Major League Baseball
PROM: Passive range of motion
RTP: Return to play
UCL: Ulnar collateral ligament
